# Impact of Airborne Exposure to PM_10_ Increases Susceptibility to *P. aeruginosa* Infection

**DOI:** 10.3390/ijerph21060722

**Published:** 2024-05-31

**Authors:** Sharon A. McClellan, Robert Wright, Farooq Muhammed, Linda D. Hazlett

**Affiliations:** Department of Ophthalmology, Visual and Anatomical Sciences, School of Medicine, Wayne State University, Detroit, MI 48201, USA; smcclell@med.wayne.edu (S.A.M.); rwrigh@med.wayne.edu (R.W.); mabdulsh@med.wayne.edu (F.M.)

**Keywords:** mice, PM_10_, cytokines, SKQ1, moxifloxacin

## Abstract

The effects of exposure to airborne particulate matter with a size of 10 μm or less (PM_10_) on C57BL/6 mouse corneas, their response to *Pseudomonas aeruginosa* (PA) infection, and the protective effects of SKQ1 were determined. C57BL/6 mouse corneas receiving PBS or SKQ1 were exposed to control (air) or PM_10_ for 2 weeks, infected, and the disease was documented by clinical score, PMN quantitation, bacterial plate count, RT-PCR and Western blot. PBS-treated, PM_10_-exposed corneas did not differ at 1 day postinfection (dpi), but exhibited earlier (3 dpi) corneal thinning compared to controls. By 3 dpi, PM_10_ significantly increased corneal mRNA levels of several pro-inflammatory cytokines, but decreased IL-10, NQO1, GR1, GPX4, and Nrf2 over control. SKQ1 reversed these effects and Western blot selectively confirmed the RT-PCR results. PM_10_ resulted in higher viable bacterial plate counts at 1 and 3 dpi, but SKQ1 reduced them at 3 dpi. PM_10_ significantly increased MPO in the cornea at 3 dpi and was reduced by SKQ1. SKQ1, used as an adjunctive treatment to moxifloxacin, was not significantly different from moxifloxacin alone. Exposure to PM_10_ increased the susceptibility of C57BL/6 to PA infection; SKQ1 significantly reversed these effects, but was not effective as an adjunctive treatment.

## 1. Introduction

Particulate matter, an air contaminant, is a general name for small atmospheric solid and liquid particles that usually vary in size (2.5–10 μm), composition, and origin [1]. The World Health Organization (WHO) air quality model shows that ambient air pollution annually causes about 4.2 million deaths, and 91% of the world’s populace lives in places where air quality exceeds the WHO guideline limits [2]. Studies performed previously have indicated that acute and chronic exposures have increased morbidity and the mortality rate globally. Specifically, in cities with elevated air pollution levels compared to those without, the individual mortality risk is greater than 26% [1]. Epidemiological evidence of adverse health effects associated with exposure to airborne particulate matter with a mean aerodynamic diameter of <10 μm (PM_10_) is one such health hazard [3]. It is associated with cardiovascular [4,5] and pulmonary diseases [6,7] and cancer [3,8]. There are also clinical data from South Korea on PM_10_ which show a correlation between exposure and increased visits for ocular diseases, including microbial keratitis [9]. In addition, a recent study in mice examined the effects of particulates in Argentina and found that they made herpes keratitis [10] worsen. Dry eye [11,12,13,14] and conjunctivitis [15,16] are also complimentary (causative or make the disease worse) to the effects of air pollution and increase susceptibility. A feature of PM_10_ exposure seems to be the induction of oxidative stress [4,7] and free radical generation [17], which are responsible for the inflammatory effects observed both in vivo and in vitro [18]. Some oxidative stress markers, among many, including reactive oxygen species (ROS) [18], myeloperoxidase expression [4], TNF-α release [19], and DNA adducts [20], have been proposed to explain the oxidative stress mechanisms induced by PM_10_. The oxidative stress effect has been attributed to PM_10_ components [21] and the large reactive surface of PM_10_ and its depositing in tissues. In the eye, there have been few studies on the effects of particulates [22,23]. One of them, an in vitro study, used a reconstructed human corneal epithelial cell model and the data suggested that ambient particulate matter >2.4 μm decreased cell viability, but did not affect the cytokines IL-6 or IL-8, and reduced the number of zonula occludens junctions [23]. A dry eye model [24] was established in BALB/c mice, which are essentially albinos that have non-pigmented eyes, relying on the topical delivery of PM_10_ (4X/day at 5 mg/mL). They found a decrease in goblet cells, microvilli on corneal surface cells, and other changes, including a higher fluorescein staining score [25].

Our in vivo model uses a standardized PM_10_, and is mechanistically focused on ROS and the nuclear factor erythroid 2-related factor 2 (Nrf2) pathway. This pathway is part of a network of inducible proteins that protect aerobic cells against the damaging effects of reactive oxygen intermediates. These cytoprotective proteins share common transcriptional regulation through the Keap1-Nrf2 pathway, which can be activated by small molecules that chemically react with critical cysteine residues of the sensor protein Kelch-like erythroid cell-derived protein with CNC homology-associated protein 1 (Keap1), leading to the stabilization of Nrf2, and ultimately to coordinating the expression of genes encoding for cytoprotective proteins [26].

The current study’s purpose is to use this model to dissect the response of mice to PM_10_, testing whether it makes them more susceptible to infection with *Pseudomonas aeuginosa* (PA), determine the signaling pathways regulating the response, and determine whether SKQ1, a mitochondrial antioxidant, is protective, particularly in combination with the antibiotic moxifloxacin.

## 2. Materials and Methods

### 2.1. Mice

Eight-week-old C57BL/6 female mice were bought from the Jackson Laboratory (Bar Harbor, ME, USA) and we housed them in accordance with the National Institutes of Health guidelines. Mice were humanely treated in compliance with both the ARVO Statement for the Use of Animals in Ophthalmic and Vision Research and the Institutional Animal Care and Use Committee of Wayne State University (IACUC 21-09-4042).

### 2.2. Whole-Body Exposure to PM_10_

Experiments in this study were performed with PM_10_ purchased from the National Institute of Standards and Technology (NIST) (Standard Reference Material (SRM) 2787). For exposure, a whole-body exposure system was used (CH Technologies, Westwood, NJ, USA) consisting of two stainless steel chambers divided into 32 compartments. Mice in one chamber were exposed to PM_10_ and in the other they received humidified, double-filtered air (control). Mice were exposed to PM_10_ or control air for 2 weeks, 3 h/day, 5 days/week at temperatures of 21–23 °C and a relative humidity of 30–70%, and rested on the weekends. PM_10_ was dispersed into the chamber using a Vilnius aerosol generator (VAG) with the in-line, real-time aerosol particle measurement device CEL-712 (Casella Microdust Pro sampler, Bedford, UK). The continuous negative feedback system was maintained at a flow rate of 4 L/min. A second measuring device, attached to the exposure chamber, recorded the concentration of particulate in the chamber every 10 s. Over the 3 h period, mice were exposed to a range of 500–1000 μg/m^3^, a range that is similar to that seen in China, India, and parts of the Middle East [27,28,29].

### 2.3. SKQ1 and Moxifloxacin Treatment

SKQ1 (BOC Sciences, Shirley, NY, USA) was used to treat PM10-exposed mice using the published dose of 7.5 μM [30]. Eyes were treated topically with 5 μL SKQ1 or PBS (control) three times on the day before the first chamber exposure and then once each day before control or PM_10_ exposure. Treatment with PBS or SKQ1 continued after infection with PA, beginning at 6 h postinfection (pi) followed by a single daily treatment at 1 and 2 days (d) pi.

In a separate set of experiments, after 2 weeks of exposure to control air or PM_10_, infected corneas were treated once per day with PBS, moxifloxacin (Lupin Pharmaceuticals, Inc., Baltimore, MD, USA) (0.25%), or SKQ1 (3.75 μM) + moxifloxacin beginning at 24 h pi.

### 2.4. Bacterial Culture and Corneal Infection

The PA strain 19660 (ATCC, Manassas, VA, USA) was grown in peptone tryptic soy broth (PTSB) medium in a rotary shaker water bath at 37 °C and 150 rpm for 18 h to reach an optical density (measured at 540 nm) between 1.3 and 1.8. The bacterial culture was centrifuged at 5500× *g* for 10 min and pellets were washed once with sterile saline, recentrifuged, resuspended, and diluted in sterile saline [31]. Mice were anesthetized with ether and placed under a stereoscopic microscope at 40× magnification. The left cornea was scratched, and 5 μL containing 1 × 10^6^ colony-forming units (CFUs)/μL of the bacterial suspension was applied topically.

### 2.5. Ocular Response to Bacterial Infection

To assess the ocular response to infection, clinical scores (*n* = 5/group/time/experiment) were designated as follows: 0 = clear or slight opacity, partially or fully covering the pupil; +1 = slight opacity, fully covering the anterior segment; +2 = dense opacity, partially or fully covering the pupil; +3 = dense opacity, covering the entire anterior segment; and +4 = corneal perforation or phthisis [32]. Each mouse was scored in a masked fashion at 1, 2, and 3 dpi for a statistical comparison and photographed (3 dpi) with a slit lamp to record disease.

### 2.6. RT-PCR

Total RNA was isolated (RNA STAT-60; Tel-Test, Friendswood, TX, USA) from 19660 infected corneas (PBS- or SKQ1-treated) exposed to control or PM_10_ (*n* = 5/group) at 3 dpi, as reported previously [33]. One microgram of each RNA sample was reverse-transcribed using Moloney murine leukemia virus (M-MLV) reverse transcriptase (Invitrogen, Carlsbad, CA, USA) to produce a cDNA template. cDNA products were diluted 1:20 with diethylpyrocarbonate (DEPC)-treated water, and a 2 μL aliquot of diluted cDNA was used for the reverse-transcription PCR (RT-PCR). A SYBR green/fluorescein PCR master mix (BioRad Laboratories, Hercules, CA, USA) and primer concentrations of 10 mM were used in a total 10 μL volume. After a preprogrammed hot start cycle (3 min at 95 °C), the parameters used for PCR amplification were 15 s at 95 °C and 60 s at 60 °C, with the cycles repeated 45 times. The fold differences in gene expression were calculated relative to naïve control, normalized to the housekeeping gene 18S rRNA, and expressed as the relative mRNA concentration ± SEM. The primer pair sequences used are shown in Table 1.

### 2.7. Western Blot

Infected corneas from control air- and PM_10_-exposed mice treated with PBS or SKQ1 were taken into ice-cold 0.1 M PBS (pH 7.4), lysed in RIPA buffer with protease and phosphatase inhibitors (SantaCruz Biotech, Dallas, TX, USA), incubated on ice for 20 min, centrifuged at 12,000× *g* at 4 °C for 10 min, and the supernatant was collected. Total protein was determined from the supernatants using a BCA protein kit (ThermoFisher Scientific, Rockford, IL, USA). In brief, samples (35 μg) were run on SDS-PAGE in Tris-glycine-SDS buffer and electro-blotted onto nitrocellulose membranes (BioRad, Herculese, CA, USA). After blocking for 1 h in 5% MTBST (Tris Buffer Saline containing 0.05% Tween 20 (TBST) and 5% nonfat milk), the membranes were probed with primary antibodies: rabbit anti-mouse TNF-α (1:1000; Cell Signaling Technology, Danvers, MA, USA), IL-1β (1:1000), CXCL2 (1:1000; Cell Signaling Technology), GPX4 (1:1000; Abcam, Waltham, MA, USA) and Nrf2 (1:1000; Cell Signaling Technology) in 5% BSA in TBST overnight at 4 °C. After three washes with TBST, membranes were incubated with HRP-conjugated anti-rabbit secondary antibody (1:2000; Cell Signaling Technology) and diluted with 5% BSA in TBST at room temperature for 2 h. Bands were developed with Supersignal West Femto Chemiluminescent Substrate (ThermoFisher Scientific), visualized using an iBright™ CL1500 Imaging System (ThermoFisher Scientific), and normalized to β-actin (1:1000; Abcam) and the intensity was quantified using Image Lab 6.1 software (BioRad). Data are shown as mean integrated density values (IDV) + SEM. The process is essentially as described previously [34].

### 2.8. Bacterial Plate Count

Infected corneas from mice exposed to control air or PM_10_ (PBS- and SKQ1-treated) were removed at 1 and 3 dpi and homogenized in 1 mL of sterile saline containing 0.25% BSA in a volume of 100 μL, serially diluted 1:10 in sterile saline containing 0.25% BSA, and the selected dilutions were then plated in triplicate on Pseudomonas isolation agar plates (Becton-Dickinson, Franklin Lakes, NJ, USA) and incubated overnight at 37 °C. Bacterial colonies were manually counted and reported as log_10_ CFU/plate +SEM.

### 2.9. P. aeruginosa and SKQ1 Killing

SKQ1 killing of *P. aeruginosa* was determined using doubled dilutions in PTSB medium. A panel of SKQ1 dilutions was prepared ranging from 30 to 0.94 μM in a 96-well microtiter plate in a volume of 200 μL per well. A 10 μL aliquot of a *P. aeruginosa* suspension (1.5 × 10^8^ CFU) was added to each well. Wells containing no SKQ1 or bacteria were used as blanks. The plate was incubated at 37 °C for 24 h with O.D. measurements at 540 nm taken every hour for 12 h and again after 24 h [35].

### 2.10. Myeloperoxidase (MPO) Assay

This assay was used to quantitate neutrophils in the infected corneas of mice that had been exposed to control air or PM_10_ (PBS- and SKQ1-treated). Briefly, individual corneas were removed at 1 and 3 dpi and homogenized in 1 mL of 50 mM phosphate buffer (pH 6.0) containing 0.5% hexadecyltrimethyl–ammonium (Sigma-Aldrich, St. Louis, MO, USA). Samples were freeze-thawed four times, centrifuged, and 100 μL of the supernatant added to 2.9 mL of 50 mM phosphate buffer containing o-dianisidine dihydrochloride (16.7 mg/mL; Sigma-Aldrich) and hydrogen peroxide (0.0005%). The changes in absorbency were monitored at 460 nm for 4 min at 30 s intervals. The slope of the line was determined for each sample and used to calculate the units of MPO/cornea. One unit of MPO activity equals approximately 2 × 10^5^ neutrophils [36].

### 2.11. Statistical Analysis

An in vivo comparison of clinical scores between two groups at each time was tested by the Mann–Whitney *U* test. A one-way ANOVA followed by Bonferroni’s multiple comparison test was used for plate counts, MPO, RT-PCR, and Western blot data. Data were considered significant at a *p* value of <0.05. All experiments were repeated at least once to ensure reproducibility and the data are shown as mean + SEM.

## 3. Results

### 3.1. Clinical Score and Slit lamp

**Figure 1:**
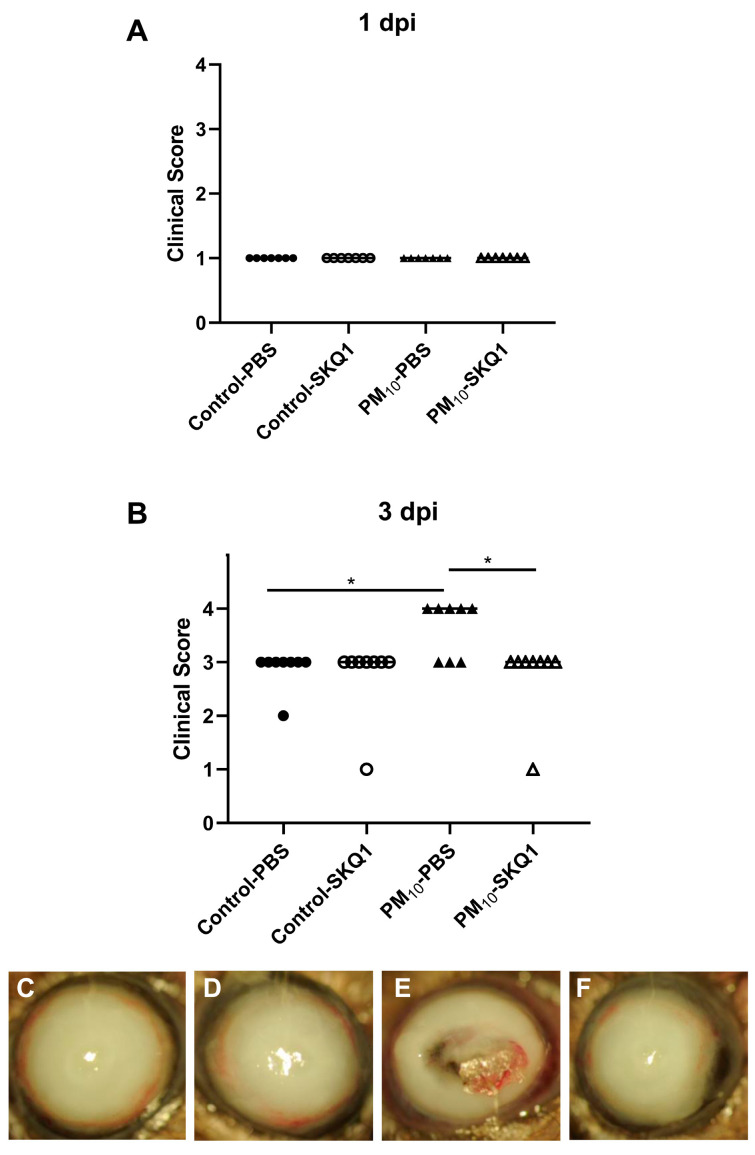
Effects of control air and PM_10_ on mouse corneas infected with strain 19660. No significant differences in clinical score are seen at 1 dpi. (**A**) At 3 dpi, the infected corneas of PBS-treated, PM_10_-exposed mice are significantly worse than the PBS-treated controls; SKQ1 treatment reduced the severity of the disease significantly (**B**). Photographs taken with a slit lamp of infected corneas at 3 dpi from PBS-treated (**C**) and SKQ1-treated (**D**) control air-exposed mice showed opacity over the entire cornea (+3), while corneas from PM_10_-exposed mice showed perforation after PBS treatment (**E**), but a similar dense opacity to control air-exposed, SKQ1-treated mice (**F**) (*n* = 10/group/time) * *p* < 0.05.

We used the clinical score and photographs taken with a slit lamp to document disease response. Figure 1A showed that there was no difference between infected eyes in the PBS- or SKQ1-treated control air and PM_10_-exposed groups at 1 dpi, where the scores were +1 for all groups. Clinical scores (Figure 1B) at 3 dpi showed a significant difference between PBS-treated control and PM_10_-exposed mice (*p* < 0.05). SKQ1 significantly reduced clinical scores in PM_10_-exposed mice (*p* < 0.05), but had no effect on disease scores from control air-exposed mice. Figure 1C–F show that the photographs of typical eyes from PA-infected mice taken with a slit lamp at 3 dpi PM_10_ exposure (PBS treatment) showed earlier perforation (Figure 1E) vs. PBS-treated control air exposure, which showed dense opacity (Figure 1C) over the entire anterior segment. Figure 1D,F show similarly dense opacity in SKQ1-treated corneas exposed to control air and PM_10_, respectively.

### 3.2. RT-PCR

**Figure 2:**
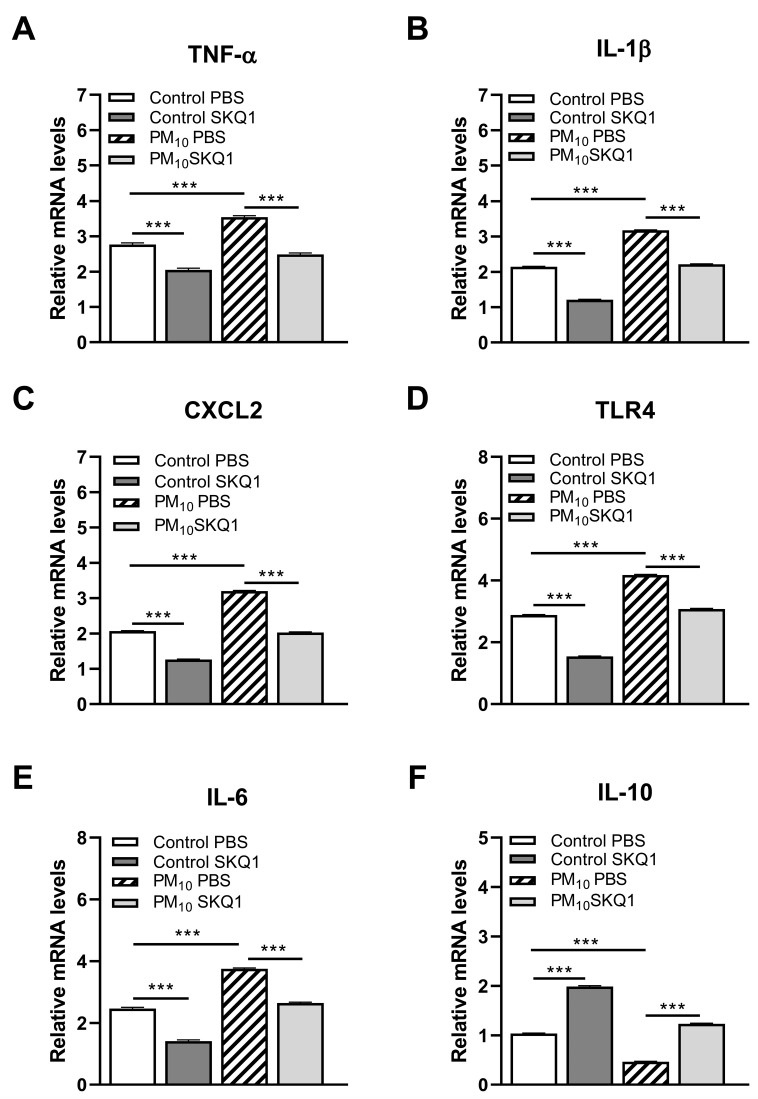
mRNA levels of corneal cytokines and chemokines after control or PM_10_ exposure and treatment with SKQ1 3 dpi. RT-PCR showed significantly elevated mRNA levels for TNF-α (**A**), IL-1β (**B**), CXCL2 (**C**), TLR4 (**D**), and IL-6 (**E**) in infected corneas after PM_10_ exposure. SKQ1 treatment reduced these levels in PM_10_- and control air-treated corneas. mRNA levels for IL-10 (**F**) were significantly reduced by PM_10_ exposure; SKQ1 treatment reversed these effects. Data are expressed as mean + SEM. (*n* = 5/group/time) *** *p* < 0.001.

**Figure 3:**
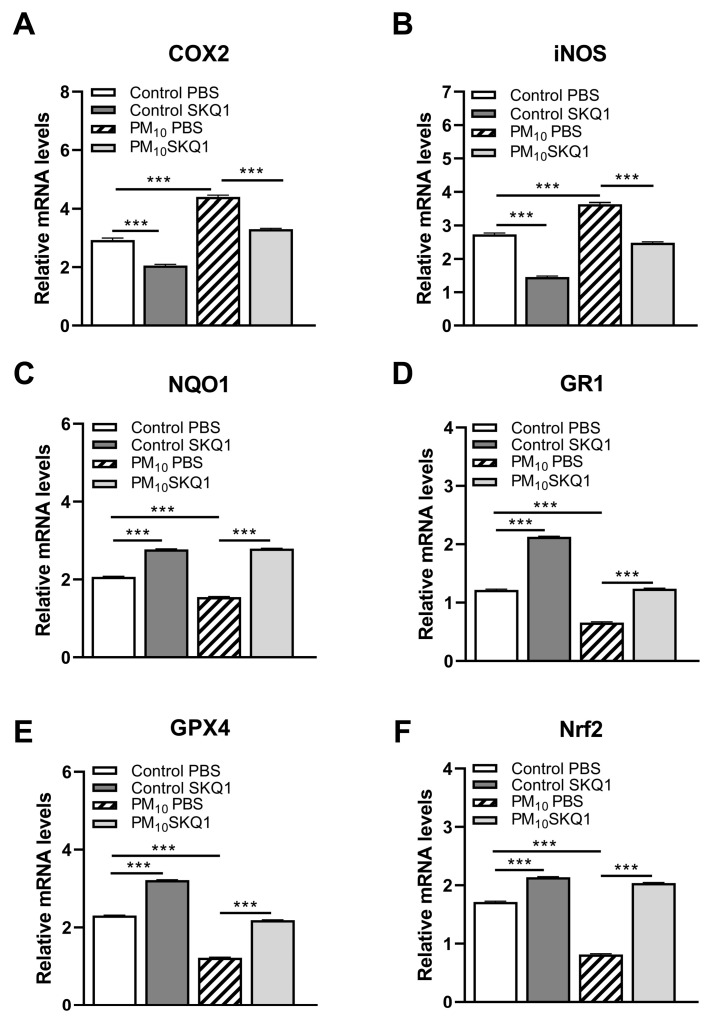
mRNA levels of corneal antioxidant-related molecules after control or PM_10_ exposure and treatment with SKQ1 3 dpi. RT-PCR showed significantly increased mRNA levels for COX2 (**A**) and iNOS (**B**) in infected corneas after PM_10_ exposure. SKQ1 treatment restored these levels in PM_10_- and control air-treated corneas. mRNA levels for NQO1 (**C**), GR1 (**D**), GPX4 (**E**), and Nrf2 (**F**) were significantly reduced by PM_10_ exposure, with SKQ1 treatment reversing these effects. Data are expressed as mean + SEM. (*n* = 5/group/time) *** *p* < 0.001.

We used RT-PCR to characterize gene expression. RT-PCR tested if PM_10_ exposure plus infection would enhance pro- or anti-inflammatory (Figure 2A–F) and oxidative stress molecules (Figure 3A–F) in C57BL/6 corneas. Exposure to PM_10_ followed by infection significantly elevated relative mRNA levels for TNF-α, IL-1β, CXCL2, TLR4, IL-6 (Figure 2A–E), COX2, and iNOS (Figure 3A,B) when compared to control air (*p* < 0.001 for all).

The mRNA levels of IL-10 (Figure 2F), NQO1, GR1, GPX4, and Nrf2 (Figure 3C–F) were significantly reduced by PM_10_ exposure of infected mice over control (*p* < 0.001 for all). SKQ1 treatment of PM_10_-exposed infected mice reversed the effects for each molecule tested and was significant (*p* < 0.001 for all).

### 3.3. Western Blot

**Figure 4:**
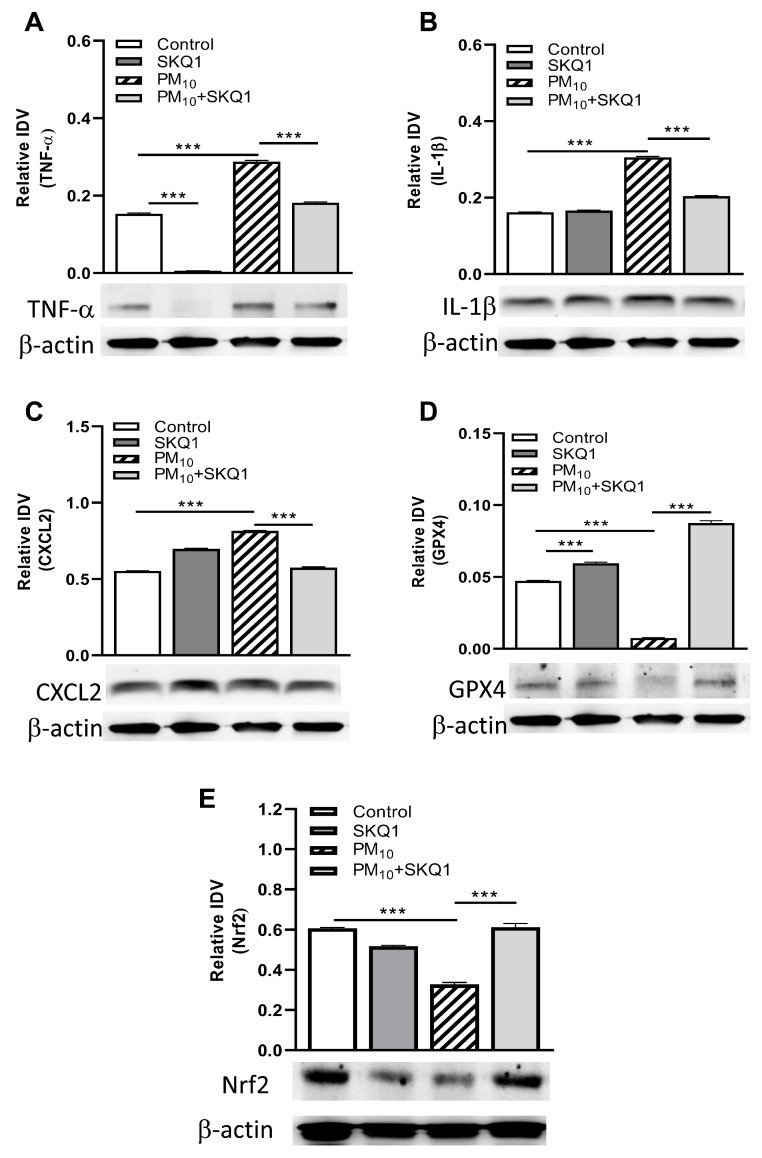
Western blot analysis of infected corneas at 3 dpi showed significantly elevated TNF-α (**A**), IL-1β (**B**), and CXCL2 (**C**) protein levels that were reduced by SKQ1 treatment. Protein levels of GPX4 (**D**) and Nrf2 (**E**) were significantly reduced after PM_10_ exposure but restored by SKQ1 treatment. Data are expressed as mean IDV + SEM. (*n* = 3/group) *** *p* < 0.001.

To validate the RT-PCR data, Western blots were used to examine the protein levels of selected cytokines and oxidative stress molecules (Figure 4A–E) after control air or PM_10_ exposure and infection. PM_10_ exposure followed by infection significantly increased the protein levels of TNF-α (A), IL-1β (B), and CXCL2 (C), and decreased GPX4 (D) and Nrf2 (E) (*p* < 0.001 for each). SKQ1 treatment after PM_10_ exposure was able to significantly reverse these effects (*p* < 0.001) for all molecules tested. When comparing the SKQ1 treatment of control air-treated infected mice, a significant reversal was seen only for TNF-α and GPX4 (*p* < 0.001) but was not observed for IL-1β, CXCL2, and Nrf2.

### 3.4. Viable Plate Count and MPO Assay

**Figure 5:**
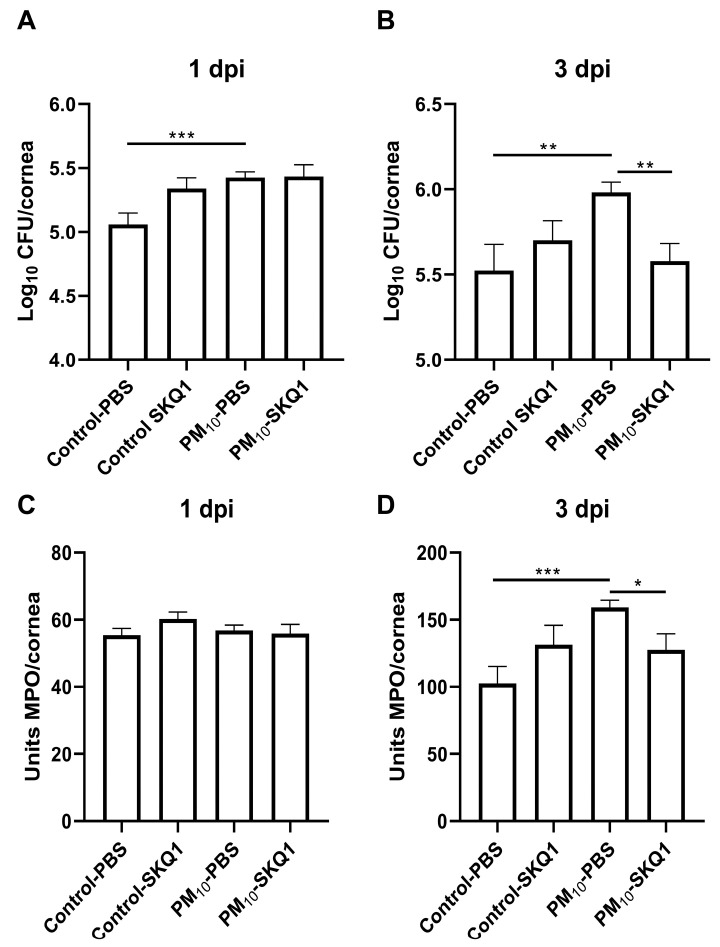
Viable bacteria and PMN quantitation. Viable bacterial plate counts were increased at both 1 (**A**) and 3 dpi (**B**) after PM_10_ exposure. SKQ1 treatment reduced this increase at 3 dpi only. The MPO assay showed a significant increase in PMN in the infected corneas of PM_10_-exposed mice at 3 dpi compared to control. The PMN number was significantly reduced by SKQ1 treatment. No differences were detected in PMN number at 1 dpi. Data expressed as mean log_10_ CFU/cornea (**A**,**B**) or mean units of MPO/cornea (**C**,**D**), + SEM. (*n* = 5/group/time) * *p* < 0.05, ** *p* < 0.01, *** *p* < 0.001.

To determine the number of viable bacteria in the cornea, we performed plate counts, and to estimate the approximate neutrophil infiltrate we measured myeloperoxidase. A viable bacterial count (Figure 5A,B) showed significantly more bacteria in the PBS-treated, PM_10_-exposed infected corneas compared to the PBS-treated controls at 1 dpi (*p* < 0.001). At 1 dpi, SKQ1 had no effect on bacterial counts in the control or PM_10_-exposed groups (Figure 4A). However, at 3 dpi, significantly more bacteria (*p* < 0.01) were seen in the PBS-treated, PM_10_-exposed infected corneas compared to the PBS-treated controls. At this time, SKQ1 treatment significantly reduced viable bacterial plate counts in the corneas of PM_10_-exposed mice when compared to the PBS-treated controls (*p* < 0.01). SKQ1 treatment had no significant effect on bacterial plate counts in control air-exposed corneas treated with PBS at 3 dpi.

An MPO assay (Figure 5C,D) did not detect any significant differences in PMN number in PBS- or SKQ1-treated corneas from infected PM_10_- vs. control air-exposed corneas at 1 dpi. However, significantly (*p* < 0.001) greater PMN number were present in the infected corneas from PBS-treated PM_10_-exposed mice compared to similarly treated controls at 3 dpi. SKQ1 treatment significantly reduced the PMN in PM_10_-exposed infected cornea (*p* < 0.05), but no difference was seen between PBS and SKQ1 treatment in the control air-exposed group.

### 3.5. SKQ1 Antibiotic Effect

**Figure 6:**
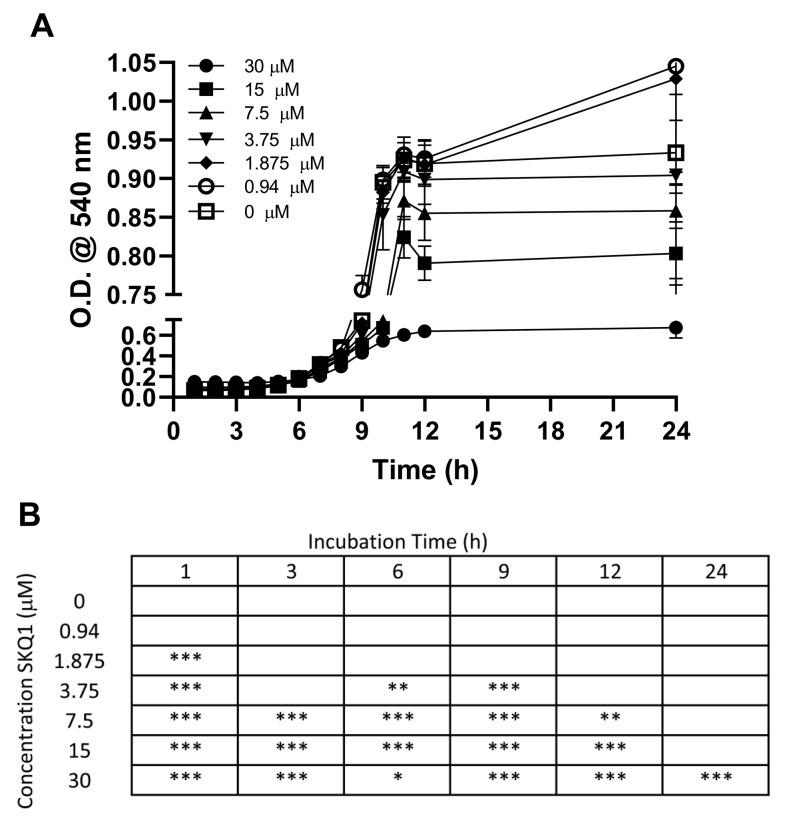
Effect of SKQ1 on growth of PA. Kinetic growth of PA in the presence of various concentrations of SKQ1 (**A**). Chart indicating the significant difference in growth between PA alone compared to growth in the presence of SKQ1 over time (**B**). (*n* = 6/dilution/time) * *p* < 0.05, ** *p* < 0.01, *** *p* < 0.001.

We tested SKQ1 for its potential antibiotic effect on PA. SKQ1 has been shown in studies to be a novel antibiotic targeting bacterial bioenergetics [35]. A killing assay was performed to test whether *P. aeruginosa*, which was not tested, was susceptible to SKQ1 killing (Figure 6A,B). Killing was determined by optical density at 540 nm of treated cultures compared to untreated control over time (Figure 6A). The statistical significance of each concentration compared to control over time is shown in Figure 6B. Overall, 30 μM was the only concentration that remained significantly reduced compared to control at 24 h (*p* < 0.001). The significant effect of 3.75 and 7.5 μM, used for treatment in these studies, was lost between 12 and 24 h, respectively.

### 3.6. Clinical Score and Slit Lamp after Moxifloxacin/SKQ1 Treatment

**Figure 7:**
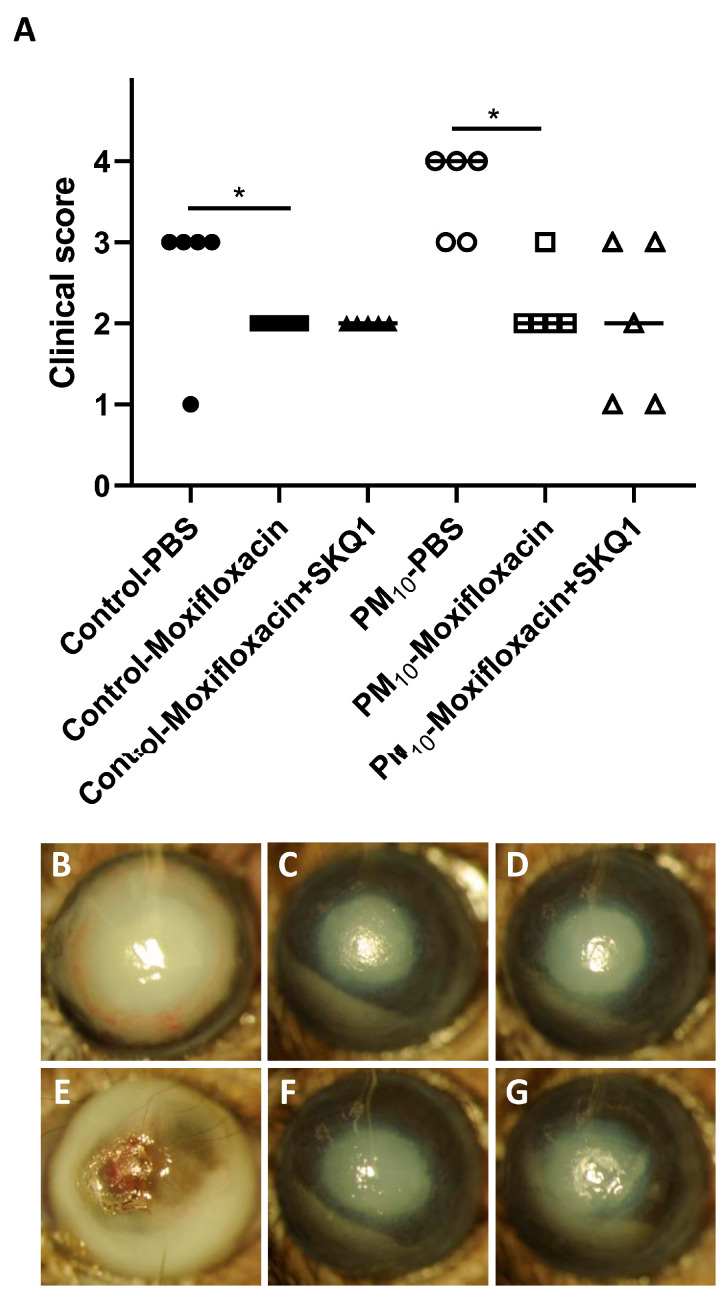
Effects of moxifloxacin +/− SKQ1 on disease. At 3 dpi, the infected corneas of Moxifloxacin-treated control and PM_10_-exposed mice were significantly better than those treated with PBS. SKQ1 treatment did not improve disease further (**A**). Photographs taken with a slit lamp of infected corneas at 3 dpi from PBS-treated control (**B**) and PM_10_-exposed mice (**E**). Control air-exposed mice showed opacity over the entire cornea (+3), while the corneas from PM_10_-exposed mice showed perforation (+4). Moxifloxacin treatment of control (**C**) and PM_10_ (**F**)-exposed corneas at 3 dpi showed reduced disease with central opacity and hypopyon. The addition of SKQ1 to Moxifloxacin treatment of control (**D**) or PM_10_ (**G**)-exposed corneas showed little improvement. (*n* = 5/group) * *p* < 0.05.

We tested disease progress after moxifloxacin/SKQ1 treatment to determine if there was an adjunctive effect of SKQ1 which added to the effect seen with the antibiotic alone. (Figure 7A) showed a significant difference between PBS-treated vs. moxifloxacin-treated control (*p* < 0.05). There was also a significant difference between PM_10_-exposed mice treated with moxifloxacin vs. PM_10_ mice treated with PBS (*p* < 0.05). Combining SKQ1 with moxifloxacin treatment showed no additional protective effect on disease scores in control air- or PM_10_-exposed mice. Figure 7B–G show slit lamp photographs of typical eyes from PA-infected mice at 3 dpi. PM_10_ exposure (PBS treatment) resulted in earlier perforation (Figure 7E) vs. PBS-treated control air exposure, which showed dense opacity (Figure 7B) over the entire anterior segment. Figure 7C,F show the disease responses in moxifloxacin control air- (Figure 7C) vs. PM_10_-exposed mice (Figure 7F), which appear similar. Figure 7D,G show moxifloxacin + SKQ1-treated mice from the control air (Figure 7D) and PM_10_ (Figure 7G) exposure groups have a similar dense opacity over the pupil. Hypopyon is present in both groups (Figure 7C,D,F,G).

### 3.7. Viable Plate Count and MPO Assay

**Figure 8:**
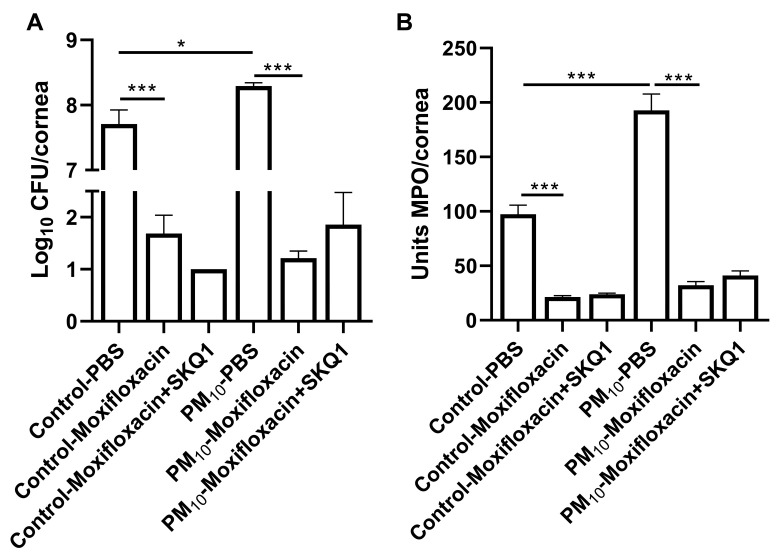
Viable bacteria and PMN quantitation after moxifloxacin +/− SKQ1 treatment. Viable bacterial plate counts were decreased at 3 dpi (**A**) after both control and PM_10_ exposure and treatment with moxifloxacin. Adding SKQ1 treatment did not further reduce plate counts. The MPO assay showed a significant decrease in PMN in the infected corneas of both control and PM_10_-exposed mice at 3 dpi after moxifloxacin treatment (**B**). Adding SKQ1 treatment to moxifloxacin had no additive effect. Data expressed as mean log_10_ CFU/cornea (**A**) or mean units of MPO/cornea (**B**) + SEM. (*n* = 5/group/time) * *p* < 0.05, *** *p* < 0.001.

We used plate count and an MPO assay to assess the number of viable bacteria present in the cornea and the approximate neutrophil number in the cornea after moxifloxacin/SKQ1 treatment. A viable bacterial count (Figure 8A) showed significantly more bacteria in the PBS-treated, PM_10_-exposed infected corneas compared to that seen in the PBS-treated control corneas at 3 dpi. (*p* < 0.05). Moxifloxacin treatment significantly reduced viable bacteria in both exposure groups (*p* < 0.001), but the addition of SKQ1 did not reduce bacterial counts further.

An MPO assay (Figure 8B) detected significantly more PMN in infected corneas from PBS-treated, PM_10_-exposed mice compared to similarly treated controls at 3 dpi (*p* < 0.001). Moxifloxacin treatment significantly reduced MPO levels in the control air (Figure 8B) and PM_10_-exposed groups (Figure 8B) (*p* < 0.001), but SKQ1 combined with Moxifloxacin did not enhance the effect in either group.

## 4. Discussion

The ocular surface is exposed to air pollutants daily, yet it is often ignored when it comes to the consequences which occur there, focusing rather on cardiovascular diseases [37,38,39,40] and cancer [41,42,43]. Major interest to date centers around countries (particularly in Asia) [44,45] which experience high levels of pollutants, which are becoming more prevalent globally. This often leads to the development of dry eye [46,47,48,49] and its consequences, which include a higher risk for microbial keratitis [50,51]. In South Korea, for example, this was documented in humans [9] who experienced increased visits to the emergency room for conjunctivitis and keratitis. This was followed by a publication from Argentina [10] which provided evidence that mice exposed to polluted air were more susceptible to herpes keratitis, as evidenced by altered immunity and worsened inflammation of the cornea. We have recently reported that pollution with PM_10_ decreases the time to perforation in bacterial infection, and examined the mechanisms involved in vitro [34]. In vivo, whole-body exposure to PM_10_ vs. control air-exposed mouse corneas showed early perforation and/or corneal thinning at 3 dpi, reduced corneal thickness, but no change in sensitivity or tears [34], and was accompanied by increased TNF-α protein levels [52].

SKQ1 has antioxidant properties and builds up in the inner mitochondrial membrane [53]. SKQ1 protects against damage caused by oxidative stress in several animal models of disease [54,55,56,57]. Recently, we found that SKQ1 is protective against PM_10_-induced oxidative damage in cultured human transformed corneal epithelial cells [34]. Correlated with this observation, an ophthalmic formulation of SKQ1 (Visomitin) has been used successfully to inhibit the pathology of anesthetic-induced dry eye syndrome after both surgery and/or lengthy general anesthesia [30]. We used this model to explore whether SKQ1, a mitochondrial antioxidant [58,59], is protective and whether it has adjuvant properties to boost the effects of the antibiotic moxifloxacin. We first compared the number of mice that received control air and/or PM_10_ ± SKQ1. We found that SKQ1 showed strong antibacterial [52] activity towards PA (a significant human pathogen) and reduced plate counts in the PM_10_-exposed group over the PBS-treated controls at 3 dpi. Others [35] have shown previously that SKQ1 is a highly effective antibiotic, with excellent killing for some Gram-positive bacteria including *Bacillus subtilis*. It was also effective against a few Gram-negative bacteria, including *Photobacterium phosphoreum* and *Rhodobacter sphaeroides*, at relatively low concentrations. SKQ1 exhibited less antibiotic activity towards *Escherichia coli* and this was due to the presence of the multidrug-resistant pump AcrAB-TolC. Mutants lacking AcrAB-TolC showed sensitivity which was similar to *B. subtilis*. The lowering of the bacterial membrane potential by SKQ1 might be involved in the mechanism of its bactericidal action, since no significant cytotoxic effect on mammalian cells was observed at bacteriostatic concentrations of SKQ1. We have not tested multidrug-resistant efflux pumps in PM_10_-exposed, PA-infected mice, but it is very likely that these pumps will provide clues to the mechanism involved in protection by SKQ1. A similar approach (proteomic analysis and RT-PCR) will be used as reported previously [60].

We next tested cytokines and chemokines to determine the host response to PM_10_ exposure followed by infection. We found that TNF-α, IL-β, CXCL2, TLR4, and IL-6 (mRNA) and selected proteins (TNF-α, IL-1β, and CXCL2) were upregulated significantly by exposure to the particulate and infection. Others [61] also have found elevated levels of ambient particulate matter PM_10_ associated with increased cardiopulmonary mortality. Circulating levels of IL-1β, IL-6, and GM-CSF were elevated in subjects exposed to high levels of PM_10_ during an episode of acute air pollution. However, they saw no change in IL-10, which we found was decreased significantly, acting, we propose, to counterbalance pro-inflammatory molecules [62,63]. Another lab reported that mice exposed to polluted air developed a severe form of herpetic keratitis with increased corneal opacity, neovascularization, and production of TNF-α, IL-1β, IFN-γ, and CCL2 [10]. GPX4 and Nrf2 (mRNA and protein) were downregulated, but the use of SKQ1 was able to restore the levels of Nrf2 and GPX4 protein significantly in our study but was not tested in theirs.

We next tested whether SKQ1 had properties similar to glycyrrhizin, which acts to boost the antibiotic treatment of PA [64]. We combined the antibiotic moxifloxacin and antioxidant SKQ1 and found that moxifloxacin alone decreased clinical scores in control air- and PM_10_-exposed infected animals significantly. Slit lamp photographs revealed that disease, as evidenced by opacity and hypopyon, was reduced significantly compared to the perforated eyes of controls. Encouraged by these data, we performed plate count and MPO assays. Again, moxifloxacin alone decreased the elevated plate count induced by PM_10_ and reduced MPO significantly. However, the addition of SKQ1 did not reduce either of them further.

## 5. Conclusions

In conclusion, we have provided evidence that SKQ1-treated, PM_10_-exposed infected eyes vs. controls showed a reduced clinical score, reduced cytokines and chemokines, reduced ROS-associated molecules, including Nrf2, and reduced plate count and MPO. We also provide evidence that SKQ1 is a potent antibacterial agent against PA, but fails to have adjuvant activity with moxifloxacin.

## Figures and Tables

**Table 1:** Nucleotide sequences of the specific primers used for PCR amplification.

Gene	Nucleotide Sequence	Primer	GenBank
*18s*	5’-GTA ACC CGT TGA ACC CCA TT-3’	F	NM_003278.3
	5’-CCA TCC AAT CGG TAG TAG CG-3′	R	
*Inf-a*	5’-ACC CTC ACA CTC AGA TCA TCT T -3′	F	NM_013693.2
	5’-GGT TGT CTT TGA GAT CCA TGC -3′	R	
*I1-1β*	5’-CGC AGC AGC ACA TCA ACA AGA GC -3′	F	NM_008361.3
	5′-TGT CCT CAT CCT GGA AGG TCC ACG -3’	R	
*Cxc12*	5′-TGT CAA TGC CTG AAG ACC CTG CC -3′	F	NM_009140.2
	5′-AAC TTT TTG ACC GCC CTT GAG AGT GG -3’	R	
*Tlr4*	5′-CCT GAC ACC AGG AAG CTT G AA -3′	F	NM_021297.2
	5′-TCT GAT CCA TGC ATT GGT AGG T -3′		
*I1-6*	5′-CAC AAG TCC GGA GAG GAG AC-3′	F	NM_031168.1
	5′-CAG AAT TGC CAT TGC ACA AC-3′	R	
*II-10*	5′-TGC TAA CCG ACT CCT TAA TGC AGG AC-3′	F	NM_010548.2
	5′-CCT TGA TTT CTG GGC CAT GCT TCT C-3′	R	
*Cox2*	5′-GCA GTT CCA GTA TCA GAA CCG CAT TG-3′	F	NM_011198.2
	5′-GAG TGA GTC CAT GTT CCA GGA GGA TG-3’	R	
*inos*	5′-TCC TCA CTG GGA CAG CAC AGA ATG-3′	F	NM_010927.3
	5′-GTG TCA TGC AAA ATC TCT CCA CTG CC-3′	R	
*Nqo1*	5′-TTC TGT GGC TTC CAG GTC TT-3′	F	NM_008706.5
	5′-TCC AGA CGT TTC TTC CAT CC-3′	R	
*Gr1*	5′-CCA CGG CTA TGC AAC ATT CG-3′	F	NM_010344.4
	5′-GAT CTG GCT CTC GTG AGG AA-3′	R	
*Gpx4*	5′-GCA ACC AGT TTG GGA GGC AGG AG-3′	F	NM_008162.4
	5′-CCT CCA TGG GAC CAT AGC GCT TC-3′	R	
*Nrf2*	5′-TGC CCC TCA TCA GGC CCA GT-3′	F	NM_010902.5
	5′-GCT CGG CTG GGA CTC GTG TT-3′	R	

F, forward: R, reverse.

## Data Availability

The original contributions presented in the study are included in the article, further inquiries can be directed to the corresponding author/s.

## References

[1] Torricelli A.A., Novaes P., Matsuda M., Alves M.R., Monteiro M.L. (2011). Ocular surface adverse effects of ambient levels of air pollution. Arq. Bras. Oftalmol..

[2] World Health Organization Air Pollution. https://www.who.int/news-room/factsheets/detail/ambient-(outdoor)-air-quality-and-health.

[3] Pope C.A., Burnett R.T., Thun M.J., Calle E.E., Krewski D., Ito K., Thurston G.D. (2002). Lung cancer, cardiopulmonary mortality, and long-term exposure to fine particulate air pollution. JAMA.

[4] Nurkiewicz T.R., Porter D.W., Barger M., Millecchia L., Rao K.M., Marvar P.J., Hubbs A.F., Castranova V., Boegehold M.A. (2006). Systemic microvascular dysfunction and inflammation after pulmonary particulate matter exposure. Environ. Health Perspect..

[5] Miller M.R., Borthwick S.J., Shaw C.A., McLean S.G., McClure D., Mills N.L., Duffin R., Donaldson K., Megson I.L., Hadoke P.W. (2009). Direct impairment of vascular function by diesel exhaust particulate through reduced bioavailability of endothelium-derived nitric oxide induced by superoxide free radicals. Environ. Health Perspect..

[6] Liu L., Poon R., Chen L., Frescura A.M., Montuschi P., Ciabattoni G., Wheeler A., Dales R. (2009). Acute effects of air pollution on pulmonary function, air-way inflammation, and oxidative stress in asthmatic children. Environ. Health Perspect..

[7] Churg A., Brauer M., del Carmen Avial-Casado M., Fortoul T.I., Wright J.L. (2003). Chronic exposure to high levels of particulate air pollution and small airway remodeling. Environ. Health Perspect..

[8] Brunekreef B., Holgate S.T. (2002). Air pollution and health. Lancet.

[9] Lee J.Y., Kim J.W., Kim E.J., Lee M.Y., Nam C.W., Chung I.S. (2018). Spatial analysis between particulate matter and emergency room visits for conjunctivitis and keratitis. Ann. Occup. Environ. Med..

[10] Sendra V.G., Tau J., Zapata G., Lasagni Vitar R.M., Illian E., Chiaradía P., Berra A. (2021). Polluted air exposure compromises corneal immunity and exacerbates inflammation in acute herpes simplex keratitis. Front. Immunol..

[11] Manisalidis I., Stavropoulou E., Stavropoulos A., Bezirtzoglou E. (2020). Environmental and health impacts of air pollution: A Review. Front. Public Health.

[12] Mo Z., Fu Q., Lyu D., Zhang L., Qin Z., Tang Q., Yin H., Xu P., Wu L., Wang X. (2019). Impacts of air pollution on dry eye disease among residents in Hangzhou, China: A case-crossover study. Environ. Pollut..

[13] Klopfer J. (1989). Effects of environmental air pollution on the eye. J. Am. Optom. Assoc..

[14] Torricelli A.A., Matsuda M., Novaes P., Braga A.L., Saldiva P.H., Alves M.R., Monteiro M.L. (2014). Effects of ambient levels of traffic-derived air pollution on the ocular surface: Analysis of symptoms, conjunctival goblet cell count and mucin 5AC gene expression. Environ. Res..

[15] Chang C.J., Yang H.H., Chang C.A., Tsai H.Y. (2012). Relationship between air pollution and outpatient visits for nonspecific conjunctivitis. Investig. Ophthalmol. Vis. Sci..

[16] Chang C.J., Yang H.H. (2020). Impact on eye health regarding gaseous and particulate pollutants. Aerosol Air Qual. Res..

[17] Li X.Y., Gilmour P.S., Donaldson K., MacNee W. (1996). Free radical activity and pro-inflammatory effects of particulate air pollution (PM10) in vivo and in vitro. Thorax.

[18] Sánchez-Pérez Y., Chirino Y.I., Osornio-Vargas Á.R., Morales-Bárcenas R., Gutiérrez-Ruíz C., Vázquez-López I., García-Cuellar C.M. (2009). DNA damage response of A549 cells treated with particulate matter (PM10) of urban air pollutants. Cancer Lett..

[19] Brown D.M., Donaldson K., Borm P.J., Schins R.P., Dehnhardt M., Gilmour P., Jimenez L.A., Stone V. (2004). Calcium and ROS-mediated activation of transcription factors and TNF-alpha cytokine gene expression in macrophages exposed to ultrafine particles. Am. J. Physiol. Lung Cell. Mol. Physiol..

[20] Valavanidis A., Vlahoyianni T., Fiotakis K. (2005). Comparative study of the formation of oxidative damage marker 8-hydroxy-2’deoxyguanosine (8-OHdG) adduct from the nucleoside 2’-deoxyguanosine by transition metals and suspensions of particulate matter in relation to metal content and redox reactivity. Free Radic. Res..

[21] Li Y.J., Takizawa H., Azuma A., Kohyama T., Yamauchi Y., Takahashi S., Yamamoto M., Kawada T., Kudoh S., Sugawara I. (2008). Disruption of Nrf2 enhances susceptibility to airway inflammatory responses induced by low-dose diesel exhaust particles in mice. Clin. Immunol..

[22] Somayajulu M., Ekanayaka S., McClellan S., Bessert D., Pitchaikannu A., Zhang K., Hazlett L.D. (2020). Airborne particulates affect corneal homeostasis and immunity. Investig. Ophthalmol. Vis. Sci..

[23] Ko R., Hayashi M., Tanaka M., Okuda T., Nishita-Hara C., Ozaki H., Uchio E. (2021). Effects of ambient particulate matter on a reconstructed human corneal epithelium model. Sci. Rep..

[24] Song S.J., Hyun S.W., Lee T.G., Park B., Jo K., Kim C.S. (2020). New application for assessment of dry eye syndrome induced by particulate matter exposure. Ecotoxicol. Environ. Saf..

[25] Li J., Tan G., Ding X., Wang Y., Wu A., Yang Q., Ye L., Shao Y. (2017). A mouse dry eye model induced by topical administration of the air pollutant particulate matter 10. Biomed. Pharmacother..

[26] Baird L., Dinkova-Kostova A.T. (2011). The cytoprotective role of the Keap1-Nrf2 pathway. Arch. Toxicol..

[27] The Economist. https://www.economist.com/the-economist-explains/2021/03/15/why-is-beijings-air-quality-so-bad-again.

[28] Express News Service. https://indianexpress.com/article/cities/delhi/delhi-weather-dust-pm10-levels-low-visibility-8611601/.

[29] Krasnov H., Katra I., Koutrakis P., Friger M.D. (2014). Contribution of dust storms to PM10 levels in an urban arid environment. J. Air Waste Manag. Assoc..

[30] Zernii E.Y., Gancharova O.S., Baksheeva V.E., Golovastova M.O., Kabanova E.I., Savchenko M.S., Tiulina V.V., Sotnikova L.F., Zamyatnin A.A., Philippov P.P. (2017). Mitochondria-Targeted antioxidant SkQ1 prevents anesthesia-induced dry eye syndrome. Oxid. Med. Cell. Longev..

[31] Kwon B., Hazlett L.D. (1997). Association of CD4+ T cell-dependent keratitis with genetic susceptibility to *Pseudomonas aeruginosa* ocular infection. J. Immunol..

[32] Hazlett L.D., Moon M.M., Strejc M., Berk R.S. (1987). Evidence for N-acetylmannosamine as an ocular receptor for *P. aeruginosa* adherence to scarified cornea. Investig. Ophthalmol. Vis. Sci..

[33] Huang X., Barrett R.P., McClellan S.A., Hazlett L.D. (2005). Silencing Toll-like receptor-9 in *Pseudomonas aeruginosa* keratitis. Investig. Ophthalmol. Vis. Sci..

[34] Somayajulu M., McClellan S.A., Wright R., Pitchaikannu A., Croniger B., Zhang K., Hazlett L.D. (2023). Airborne exposure of the cornea to PM_10_ induces oxidative stress and disrupts Nrf2 mediated anti-oxidant defenses. Int. J. Mol. Sci..

[35] Nazarov P.A., Kotova E.A., Skulachev V.P., Antonenko Y.N. (2019). Genetic variability of the AcrAB-TolC multidrug efflux pump underlies SkQ1 resistance in gram-negative bacteria. Acta Nat..

[36] Williams R.N., Paterson C.A., Eakins K.E., Bhattacherjee P. (1982). Quantification of ocular inflammation: Evaluation of polymorphonuclear leucocyte infiltration by measuring myeloperoxidase activity. Curr. Eye Res..

[37] Rajagopalan S., Al-Kindi S.G., Brook R.D. (2018). Air pollution and cardiovascular disease: JACC State-of-the-Art Review. J. Am. Coll. Cardiol..

[38] de Bont J., Jaganathan S., Dahlquist M., Persson Å., Stafoggia M., Ljungman P. (2022). Ambient air pollution and cardiovascular diseases: An umbrella review of systematic reviews and meta-analyses. J. Intern. Med..

[39] Krittanawong C., Qadeer Y.K., Hayes R.B., Wang Z., Virani S., Thurston G.D., Lavie C.J. (2023). PM2.5 and cardiovascular health risks. Curr. Probl. Cardiol..

[40] Zhang S., Qian Z.M., Chen L., Zhao X., Cai M., Wang C., Zou H., Wu Y., Zhang Z., Li H. (2023). Exposure to air pollution during pre-hypertension and subsequent hypertension, cardiovascular disease, and death: A trajectory analysis of the UK Biobank Cohort. Environ. Health Perspect..

[41] Huang Y., Zhu M., Ji M., Fan J., Xie J., Wei X., Jiang X., Xu J., Chen L., Yin R. (2021). Air pollution, genetic factors, and the risk of lung cancer: A prospective study in the UK Biobank. Am. J. Respir. Crit. Care Med..

[42] Zare Sakhvidi M.J., Lequy E., Goldberg M., Jacquemin B. (2020). Air pollution exposure and bladder, kidney and urinary tract cancer risk: A systematic review. Environ. Pollut..

[43] Valavanidis A., Vlachogianni T., Fiotakis K., Loridas S. (2013). Pulmonary oxidative stress, inflammation and cancer: Respirable particulate matter, fibrous dusts and ozone as major causes of lung carcinogenesis through reactive oxygen species mechanisms. Int. J. Environ. Res. Public Health.

[44] Junaid M., Syed J.H., Abbasi N.A., Hashmi M.Z., Malik R.N., Pei D.S. (2018). Status of indoor air pollution (IAP) through particulate matter (PM) emissions and associated health concerns in South Asia. Chemosphere.

[45] Dutta A., Jinsart W. (2022). Air pollution in Delhi, India: It’s status and association with respiratory diseases. PLoS ONE.

[46] Lu C.W., Fu J., Liu X.F., Cui Z.H., Chen W.W., Guo L., Li X.L., Ren Y., Shao F., Chen L.N. (2023). Impacts of air pollution and meteorological conditions on dry eye disease among residents in a northeastern Chinese metropolis: A six-year crossover study in a cold region. Light. Sci. Appl..

[47] Alves M., Asbell P., Dogru M., Giannaccare G., Grau A., Gregory D., Kim D.H., Marini M.C., Ngo W., Nowinska A. (2023). TFOS Lifestyle Report: Impact of environmental conditions on the ocular surface. Ocul. Surf..

[48] Kim Y., Choi Y.H., Kim M.K., Paik H.J., Kim D.H. (2020). Different adverse effects of air pollutants on dry eye disease: Ozone, PM_2.5_, and PM_10_. Environ. Pollut..

[49] Brito-Zerón P., Retamozo S., Ramos-Casals M. (2023). Sjögren syndrome. Med. Clin..

[50] Tsai T.Y., Adiyabazar D., Hsiao C.H., Pan L.Y., Chen S.Y., Tsai Y.J., Chen C.B., Chung W.H., Ma D.H. (2023). Microbial keratitis in patients with Stevens-Johnson syndrome and toxic epidermal necrolysis: Experience from a tertiary centre in Taiwan. Cornea.

[51] Somani S.N., Ronquillo Y., Moshirfar M. (2024). Acanthamoeba Keratitis. StatPearls [Internet].

[52] Somayajulu M., McClellan S.A., Muhammed F., Wright R., Hazlett L.D. (2023). PM_10_ and *Pseudomonas aeruginosa*: Effects on corneal epithelium. Front. Cell Infect. Microbiol..

[53] Skulachev V.P. (2013). Cationic antioxidants as a powerful tool against mitochondrial oxidative stress. Biochem. Biophys. Res. Commun..

[54] Demianenko I.A., Vasilieva T.V., Domnina L.V., Dugina V.B., Egorov M.V., Ivanova O.Y., Ilinskaya O.P., Pletjushkina O.Y., Popova E.N., Sakharov I.Y. (2010). Novel mitochondria-targeted antioxidants, “Skulachev-ion” derivatives, accelerate dermal wound healing in animals. Biochemistry.

[55] Skulachev V.P. (2011). SkQ1 treatment and food restriction—Two ways to retard an aging program of organisms. Aging.

[56] Genrikhs E.E., Stelmashook E.V., Popova O.V., Kapay N.A., Korshunova G.A., Sumbatyan N.V., Skrebitsky V.G., Skulachev V.P., Isaev N.K. (2015). Mitochondria-targeted antioxidant SkQT1 decreases trauma-induced neurological deficit in rat and prevents amyloid-β-induced impairment of long-term potentiation in rat hippocampal slices. J. Drug Target..

[57] Kezic A., Spasojevic I., Lezaic V., Bajcetic M. (2016). Mitochondria-targeted antioxidants: Future perspectives in kidney ischemia reperfusion injury. Oxid. Med. Cell. Longev..

[58] Huang B., Zhang N., Qiu X., Zeng R., Wang S., Hua M., Li Q., Nan K., Lin S. (2023). Mitochondria-targeted SkQ1 nanoparticles for dry eye disease: Inhibiting NLRP3 inflammasome activation by preventing mitochondrial DNA oxidation. J. Control. Release.

[59] Nazarov P.A., Osterman I.A., Tokarchuk A.V., Karakozova M.V., Korshunova G.A., Lyamzaev K.G., Skulachev M.V., Kotova E.A., Skulachev V.P., Antonenko Y.N. (2017). *Mitochondria*-targeted antioxidants as highly effective antibiotics. Sci. Rep..

[60] Hazlett L.D., Ekanayaka S.A., McClellan S.A., Francis R. (2019). Glycyrrhizin use for multi-drug resistant *Pseudomonas aeruginosa*: In vitro and in vivo studies. Investig. Ophthalmol. Vis. Sci..

[61] van Eeden S.F., Tan W.C., Suwa T., Mukae H., Terashima T., Fujii T., Qui D., Vincent R., Hogg J.C. (2001). Cytokines involved in the systemic inflammatory response induced by exposure to particulate matter air pollutants (PM(10)). Am. J. Respir. Crit. Care Med..

[62] Foldenauer M.E., McClellan S.A., Berger E.A., Hazlett L.D. (2013). Mammalian target of rapamycin regulates IL-10 and resistance to *Pseudomonas aeruginosa* corneal infection. J. Immunol..

[63] Hazlett L.D., Jiang X., McClellan S.A. (2014). IL-10 function, regulation, and in bacterial keratitis. J. Ocul. Pharmacol. Ther..

[64] Somayajulu M., McClellan S.A., Bessert D.A., Pitchaikannu A., Hazlett L.D. (2022). Ocular effects of glycyrrhizin at acidic and neutral pH. Front. Cell Infect. Microbiol..

